# Posterior sternoclavicular joint dislocation in a young male: a case report

**DOI:** 10.11604/pamj.2024.47.138.28888

**Published:** 2024-03-26

**Authors:** William Ngatchou, Michèle Ngassa Fosso, Virginie Guimfacq Djumegue, Ion-Rares Surdeanu, Patrice Jissendi, Pierre Youatou Towo

**Affiliations:** 1 Department of Emergency Medicine, University Hospital UCH Saint-Pierre, Brussels, Belgium,; 2 Department of Surgery and Specialties, Faculty of Medicine and Pharmaceutical Sciences, Douala University, Douala, Cameroon,; 3 Department of Gastroenterology, University Hospital Brugmann, Brussels, Belgium,; 4 Department of Cardiology, University Hospital Delta, Brussels, Belgium,; 5 Department of Radiology, University Hospital Saint-Pierre, Brussels, Belgium

**Keywords:** Posterior luxation, clavicle, sternoclavicular, impact injury, case report

## Abstract

The traumatic dislocation of the posterior sternoclavicular joint is a serious injury with possibly severe complications and therefore has to be managed with the greatest caution. We report the case of a young male with a posterior dislocation of the medial clavicle with compression of the brachiocephalic artery as well as the esophagus. Open reduction and placement of a wire cerclage were performed with a good postoperative outcome.

## Introduction

The posterior sternoclavicular dislocation (PSCD) is a serious injury, mostly caused by high-impact trauma such as motorcycle accidents, athletic injuries, and falls [1]. Because of its proximity to the superior mediastinal structures comprising the great vessels including the aorta and its principal branches, the superior vena cava and the jugular vein, the trachea, the esophagus, the recurrent laryngeal nerve, and the lunges, possibly bringing forth complications as severe as brachial plexopathy [2], respiratory compromise, pneumothorax, dysphagia, vascular injury, and even death [3,4]. Therefore, a prompt diagnosis is, although often difficult, crucial to achieve the best possible outcome. Though chest X-rays are commonly used for initial diagnosis, a computed tomography (CT) scan is required for confirmation as well as (pre-operative) management. The therapeutic method of choice is a timely closed or open reduction.

## Patient and observation

**Patient information:** a 20-year-old patient presented to the emergency room after having endured a sporting accident while playing hockey, where he was squished between a playmate and the tribune. The patient claimed having heard a cracking noise in the area of his right clavicle and complained of pain and immobility in the same area as well as respiratory distress and swallowing difficulties.

**Clinical findings:** clinically he presented with a slight deformation of the right clavicle, no bony crepitus, and a normal neurovascular examination. Upon arrival, the patient was fully conscious with a Glasgow Coma Scale of 15, hemodynamically stable with a palpable axillary and radial pulse on the right, and breathing spontaneously.

**Figure 1:**
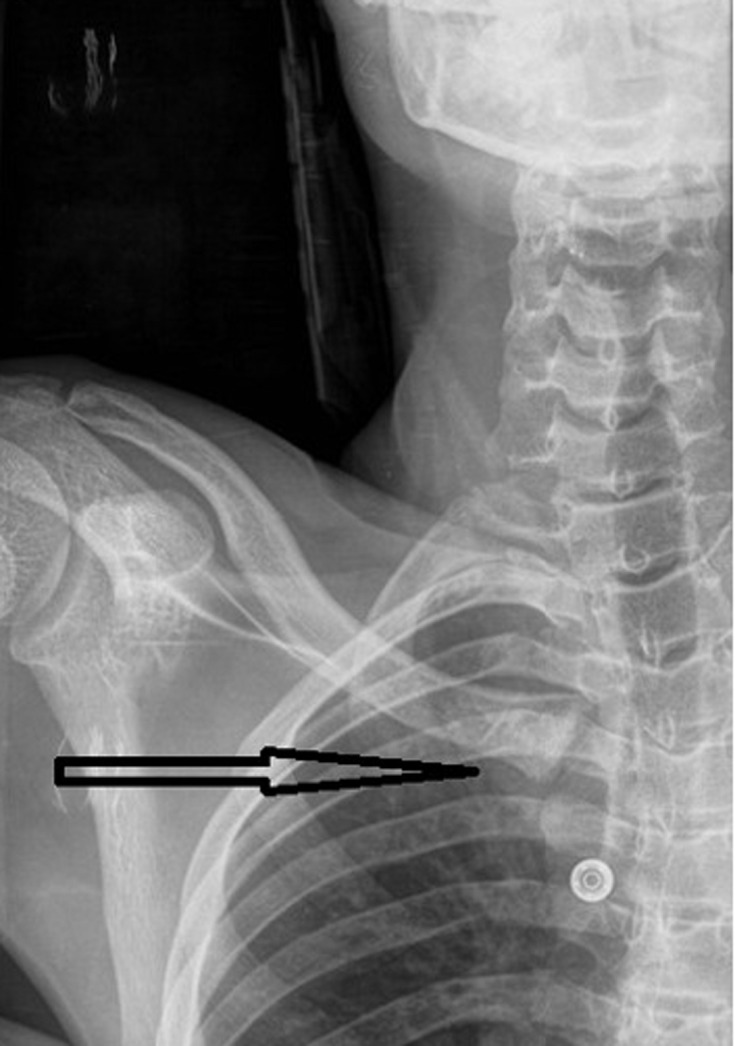
inferior displacement of the right clavicle

**Figure 2:**
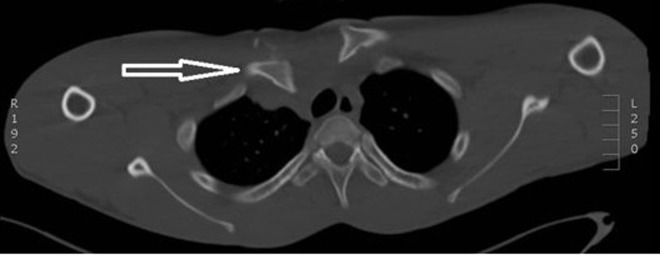
computed tomography scan view of the sternoclavicular dislocation

**Diagnostic assessment:** the initial radiograph of the right shoulder showed a slight inferior displacement of the right clavicle, a pneumothorax could be ruled out (Figure 1). Together with the conspicuous clinic, a suspicion of right clavicular displacement was imposed and promptly confirmed as a posterior sternoclavicular luxation with posterior displacement of the medial clavicle in the following CT scan (Figure 2).

**Figure 3:**
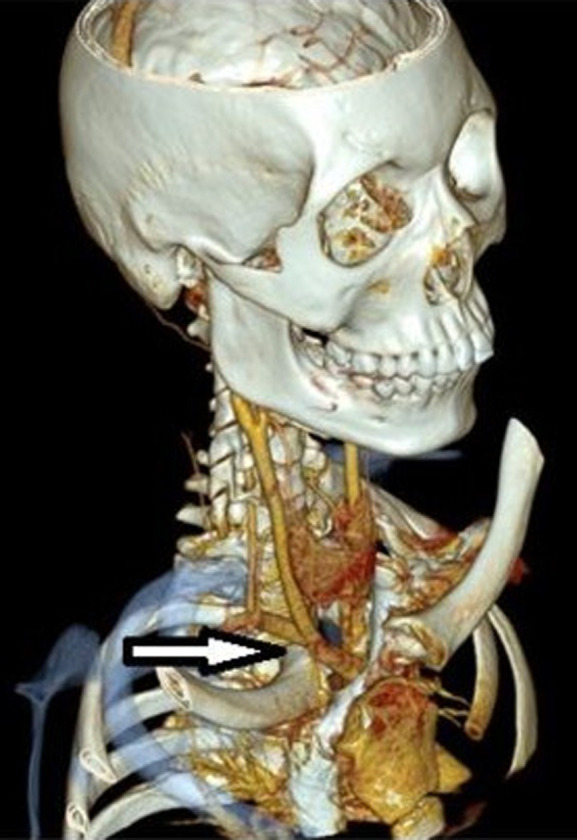
proximity of the brachiocephalic artery

Due to persistent respiratory distress, swallowing difficulties, and acute proximity to the brachiocephalic artery as unveiled in the CT angiography (Figure 3), the patient was immediately hospitalized and it was decided to directly opt for surgical rather than closed reduction.

**Therapeutic intervention:** the surgery was performed in a beach chair position in the presence of an orthopedic as well as a cardiothoracic surgeon. The clavicle was reduced with Davier´s forceps and a 0.8 mm stainless steel wire was placed in a figure-of-eight-shaped manner in the medial extremity of the clavicle as well as the manubrium of the sternum for stabilization. After its placement, articular stability was clinically verified and a postoperative chest X-ray showed full restoration of the normal anatomy of the thoracic cage and no evidence of pneumothorax.

**Figure 4:**
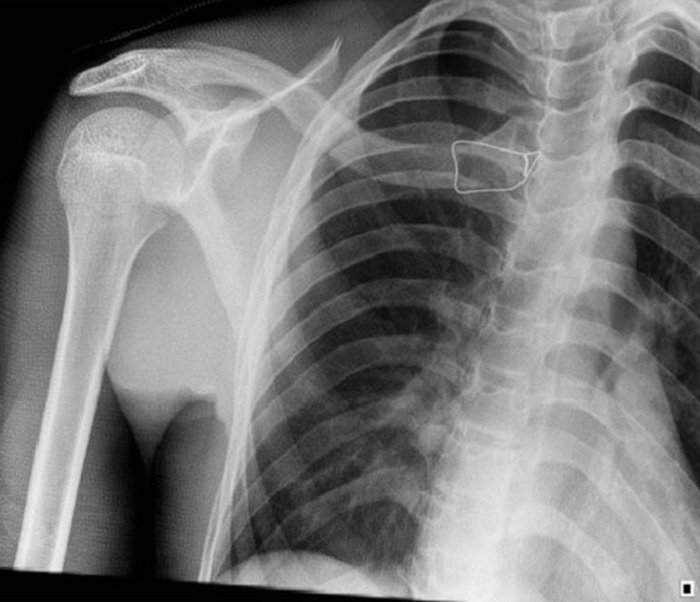
four weeks postoperative X-ray

The patient was mobilized with a Desault´s bandage for six weeks. All respiratory and swallowing difficulties had disappeared after the intervention and the patient presented with a good postoperative evolution at his 4- and 6-week follow-up. After 6 weeks, the Desault´s bandage was removed and a control X-ray once again showed no abnormalities (Figure 4).

**Follow-up and outcomes:** the patient showed a full restoration in range of motion. Unfortunately, the patient did not turn up for any of the scheduled long-term check-ups. Therefore, a constant score [5], for description and comparison of functionality could not be evaluated.

**Informed consent:** the patient has given informed consent.

## Discussion

The sternoclavicular joint is a saddle-type synovial joint between the medial end of the clavicle, the clavicular notch of the manubrium, and the upper part of the first costal cartilage. However, only a small part of the medial clavicle interacts with the manubrium. Because of said incongruousness, the joint proves to be unstable and is principally supported by a fibrous capsule, an intraarticular disc, anterior and posterior sternoclavicular ligaments as well as inter- and costo-clavicular ligaments.

The PSCD is a rare condition of which the first case was reported by Sir. Cooper in 1824 and since then only a little over one hundred cases have been described [6].

In the absence of any clinical and radiographical signs of mediastinal injury, closed reduction under general anesthesia is the treatment of choice in acute PSCD [7]. This procedure is always to be performed in the presence of a cardiothoracic surgeon because of the risk of damage to the underlying mediastinal structures [4]. In case of clinical signs of mediastinal involvement, open reduction, and internal fixation are the therapy of choice. To obtain a lasting stabilization of the joint while preserving shoulder mobility, which excludes an arthrodesis, various fixation techniques have been described in literature reaching from hard-wire cerclages, synthetic ligament, and bone sutures to tendon-grafts and fascia lata flaps [8]. Though the optimal is yet to be found, fixation with orthopedic pins and wires such as Kirschner wires or Steinmann pins is obsolete and should be avoided because of their risk of migration towards the underlying vital structures [8,9]. Further resection of the medial end of the clavicle without stabilization cannot be recommended because of the poor postoperative outcome for the patient regarding freedom of movement and pain [10].

## Conclusion

Since the tendon graft figure-of-eight reconstruction using a palmaris, plantaris, or semitendinosus tendon, would have required an additional surgical approach in this young patient, we chose fixation with bone sutures. Our technique with a figure-of-eight cerclage wire, ensures a stronger posterior reinforcement of the joint than conventional suture material and allows for natural healing of the local ligaments and capsule.
